# Association Between HAVOC Score and New-Onset Atrial Fibrillation in Patients With ST-Segment Elevation Myocardial Infarction

**DOI:** 10.31083/RCM47061

**Published:** 2026-02-25

**Authors:** Shuang Zhou, Dongdong Cai, Zhiwen Wang, Xinchun Gao, Wen Lu

**Affiliations:** ^1^The Xuzhou Clinical College of Xuzhou Medical University, 221000 Xuzhou, Jiangsu, China; ^2^Department of Cardiology, The Affiliated Shuyang Hospital of Xuzhou Medical University, 223600 Suqian, Jiangsu, China; ^3^Department of Cardiology, Xuzhou Central Hospital, 221002 Xuzhou, Jiangsu, China

**Keywords:** cardiovascular disease, HAVOC score, atrial fibrillation, ST-segment elevation myocardial infarction

## Abstract

**Background:**

The HAVOC score is an emerging tool for estimating the risk of atrial fibrillation (AF), which has attracted growing interest. However, the use of the HAVOC score to predict in-hospital new-onset AF (NOAF) among patients with ST-segment elevation myocardial infarction (STEMI) remains unclear. Therefore, this study aimed to examine whether the HAVOC score is associated with NOAF during the index hospitalization following primary percutaneous coronary intervention (PCI) in patients with a STEMI.

**Methods:**

We studied a consecutive cohort of patients presenting with STEMI from January 2023 to March 2025. After primary PCI, each participant underwent continuous electrocardiogram monitoring for at least 72 hours. The HAVOC score was calculated based on hypertension, age ≥75 years, valvular heart disease, peripheral vascular disease, obesity, and heart failure.

**Results:**

In total, 725 patients were analyzed, with a mean age of 63.37 ± 13.16 years; of whom 72.97% were male. During the hospital stay, 70 patients (9.66%) experienced NOAF. Multivariate logistic regression analysis showed that the HAVOC score (odds ratio (OR) = 1.42, 95% confidence interval (CI): 1.28–1.59) was independently associated with NOAF. Restricted cubic spline (RCS) analysis revealed a linear dose–response relationship between the HAVOC score and NOAF (*p* for overall <0.001). Integrating left ventricular ejection fraction (LVEF) and the presence of left anterior descending artery stenosis enhanced the discriminatory ability of the HAVOC score for identifying NOAF (net reclassification index [NRI] = 0.353, 95% CI: 0.114–0.592; *p* = 0.004) and improved integrated discrimination (0.024, 95% CI: 0.006–0.041; *p* = 0.008).

**Conclusions:**

Higher HAVOC scores were independently linked to the occurrence of in-hospital NOAF among STEMI patients following PCI. NOAF risk increased with the HAVOC score, consistent with a linear dose–response across the score spectrum.

## 1. Introduction

ST-segment elevation myocardial infarction (STEMI) is a common yet highly lethal presentation associated with cardiovascular disease [1]. Although there has been substantial progress in early recognition, reperfusion techniques, and secondary prevention, complications continue to occur both during the index admission and following hospital discharge [1, 2]. Atrial fibrillation (AF) is often precipitated by acute ischemic injury and necrosis of myocardial tissue during STEMI, which alter the electrophysiological properties of the heart [3, 4]. Previous studies have indicated that the incidence of new-onset AF (NOAF) in STEMI patients can be as high as 20% and is closely associated with poor prognosis [5, 6]. Currently, there is controversy regarding the optimal prevention and treatment strategies for NOAF in STEMI patients during their hospitalization [7]. Therefore, identifying the risk factors associated with NOAF, accurately stratifying high-risk patients, and formulating effective preventive and therapeutic approaches are crucial for improving the clinical outcomes of STEMI patients.

Tools designed to predict NOAF in the STEMI population are still scarce. The HAVOC Score—comprising Hypertension, Age ≥75, Valvular heart disease, Peripheral vascular disease, Obesity, Heart failure, and Coronary artery disease—has emerged as a tool for predicting the occurrence of AF, and has attracted increasing interest in recent years [8, 9, 10, 11]. Compared with other AF risk scores, the HAVOC Score places greater emphasis on predicting the initial occurrence of AF. The HAVOC Score integrates seven clinical parameters and has been extensively validated in stroke patients with NOAF [8, 9, 10, 11]. The improved accuracy of HAVOC in AF prediction may be attributed to the close association between the components of the HAVOC Score and the pathophysiological mechanisms of AF, such as inflammation and myocardial fibrosis [12, 13, 14]. However, whether HAVOC is linked to in-hospital NOAF among STEMI patients has not been definitively established. We sought to evaluate the association between the HAVOC Score and NOAF occurring during the index hospitalization following primary percutaneous coronary intervention (PCI) in STEMI patients in order to determine whether the HAVOC score would provide an additional risk stratification tool that enables more accurate identification of high-risk patients and facilitates individualized risk assessment.

## 2. Materials and Methods

### 2.1 Study Population

**Fig. 1.**
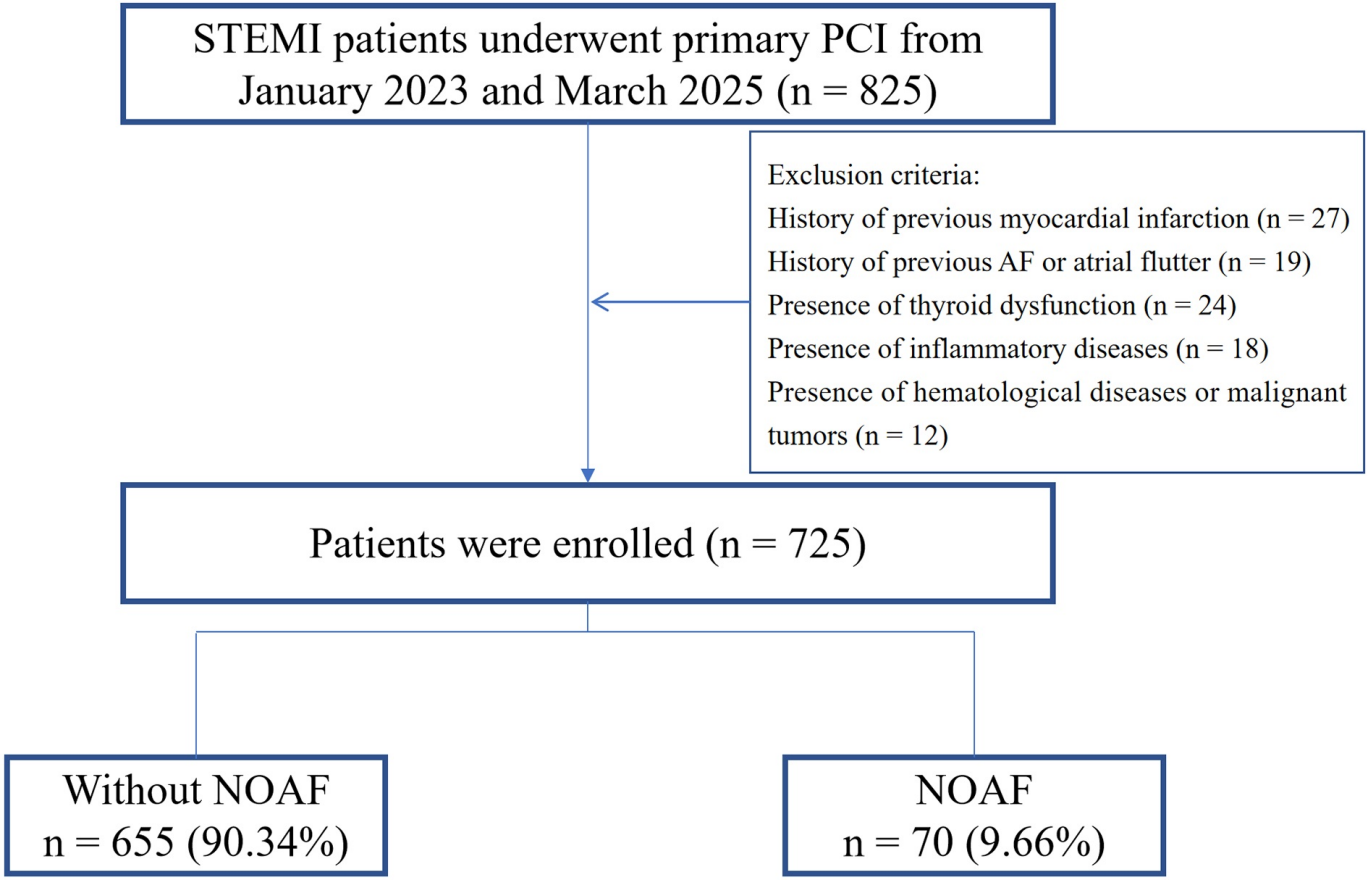
**Study flowchart**. STEMI, ST-segment elevation myocardial infarction; NOAF, new-onset atrial fibrillation; AF, atrial fibrillation; PCI, percutaneous coronary intervention.

This study retrospectively enrolled STEMI [15] patients who received treatment at the Xuzhou Central Hospital between January 2023 and March 2025. The inclusion criteria were as follows: (1) Successful completion of primary PCI (thrombolysis in myocardial infarction [TIMI]= 3). (2) All patients underwent continuous electrocardiogram (ECG) monitoring (telemetry) for at least 72 hours post-PCI. (3) Complete clinical data were available. The exclusion criteria were: (1) History of previous myocardial infarction (MI). (2) History of previous AF or atrial flutter. (3) Presence of thyroid dysfunction. (4) Presence of inflammatory diseases. (5) Presence of hematological diseases or malignant tumors. The study protocol was reviewed and approved by the Institutional Review Board (IRB) of the Xuzhou Central Hospital (XZXY-LK-20250317-0034), and all procedures were conducted in accordance with the ethical principles of the Declaration of Helsinki. This retrospective analysis involved no harm to the participants; therefore, the IRB authorized a waiver of informed consent. In total, 725 individuals were ultimately enrolled in the present study (Fig. 1).

### 2.2 Data Collection

All research data were systematically collected from the electronic medical record system. The collected information included: Demographic characteristics and the use of medications. Comorbidities including heart failure (HF), hypertension, diabetes mellitus, peripheral vascular disease, and valvular heart disease were recorded. Laboratory tests including electrolytes, lipid profile, high-sensitivity C-reactive protein (hs-CRP), troponin I (TNI), and N-terminal pro-brain natriuretic peptide (NT-proBNP) were included. Information about the infarct-related artery (IRA) was determined based on the results of coronary angiography. The HAVOC Score was calculated according to hypertension (2 points), age ≥75 years (2 points), valvular heart disease (2 points), peripheral vascular disease (1 point), obesity (body mass index (BMI) >30 kg/m^2^) (1 point), HF (4 points), and coronary artery disease (CAD) (2 points) [8, 9, 10, 11]. Considering that all STEMI patients inherently have CAD, we excluded the CAD component when calculating the HAVOC score in this study. AF was diagnosed by ECG when P waves were absent and replaced by irregular fibrillatory waves with variable morphology, amplitude, and duration, accompanied by completely irregular RR intervals, documented on either a single-lead tracing lasting ≥30 seconds or a standard 12-lead ECG [16]. NOAF was defined as the first documented episode of AF occurring during hospitalization in patients who had no previous history of AF.

### 2.3 Statistical Analysis

SPSS version 27.0 (IBM, Chicago, IL, USA) and R 4.4.3 (Lucent Technologies, New Jersey, USA) were used to perform statistical analyses. The normality of continuous variables was assessed using the Kolmogorov–Smirnov test. Continuous data were expressed as mean ± standard deviation (SD) or as median (interquartile range, IQR), whereas categorical variables were presented as counts and percentages. Differences between groups were evaluated using the independent samples *t*-test for normally distributed data and the Mann–Whitney U test for non-normally distributed data. Comparisons of categorical variables were conducted using the χ^2^ test or Fisher’s exact test, as appropriate. Variables with *p* < 0.05 screened by univariate logistic regression analysis were incorporated into the multivariate regression model together with traditional risk factors using the stepwise method. We assessed the HAVOC Score’s ability to predict NOAF using receiver operating characteristic (ROC) analysis, reporting the area under the curve (AUC). The optimal threshold was identified by maximizing the Youden index. We used the net reclassification index (NRI), and integrated discrimination improvement (IDI) to assess the additional discriminatory ability of HAVOC for NOAF. A restricted cubic spline (RCS) model was fitted to characterize the continuous, dose–response relationship between HAVOC and in-hospital NOAF risk. All statistical analyses with *p* < 0.05 were considered to be statistically significant.

## 3. Results

### 3.1 Baseline Data Comparison Between Groups

In total, 725 patients were analyzed, with a mean age of 63.37 ± 13.16 years; 72.97% were male. During the hospital stay, 70 patients (9.66%) experienced NOAF. The results of the baseline characteristics analysis demonstrated that the patients with NOAF exhibited significantly higher values in terms of age, HAVOC Score, NT-proBNP, and hs-CRP (*p* < 0.05). The left ventricular ejection fraction (LVEF) in the patients with NOAF was significantly lower (*p* < 0.05). Among the categorical variables, the proportion of patients with HF and the Killip class in patients with NOAF were significantly higher than those in the group of patients without NOAF (*p* < 0.05) (Table 1).

**Table 1.** **Patient characteristics**.

Variables		Total (n = 725)	Non-NOAF (n = 655)	NOAF (n = 70)	*p*
Age, years		63.37 ± 13.16	62.14 ± 12.86	74.90 ± 9.91	<0.001
Male, n (%)		529 (72.97)	484 (73.89)	45 (64.29)	0.085
BMI, kg/m^2^		24.63 ± 3.54	24.60 ± 3.43	24.97 ± 4.51	0.503
Heart rate, bpm		79.40 ± 13.92	79.18 ± 13.89	81.50 ± 14.14	0.185
SBP, mmHg		126.68 ± 21.39	126.78 ± 20.70	125.73 ± 27.19	0.755
DBP, mmHg		78.75 ± 13.54	78.97 ± 13.46	76.71 ± 14.22	0.186
Smoking, n (%)		326 (44.97)	297 (45.34)	29 (41.43)	0.531
HF, n (%)		375 (51.72)	320 (48.85)	55 (78.57)	<0.001
Valvular disease, n (%)		121 (16.69)	106 (16.18)	15 (21.43)	0.263
PVD, n (%)		556 (76.69)	501 (76.49)	55 (78.57)	0.695
Hypertension, n (%)		300 (41.38)	264 (40.31)	36 (51.43)	0.072
Diabetes, n (%)		168 (23.17)	148 (22.60)	20 (28.57)	0.260
Stroke, n (%)		84 (11.59)	75 (11.45)	9 (12.86)	0.727
HAVOC Score		4.48 ± 2.74	4.25 ± 2.64	6.71 ± 2.70	<0.001
TC, mmol/L		4.41 ± 1.05	4.43 ± 1.05	4.26 ± 1.02	0.191
Triglycerides, mmol/L		1.47 ± 0.93	1.46 ± 0.90	1.63 ± 1.20	0.155
LDL-C, mmol/L		2.75 ± 0.90	2.77 ± 0.91	2.56 ± 0.79	0.060
HDL-C, mmol/L		1.05 ± 0.28	1.06 ± 0.28	1.00 ± 0.26	0.080
TNI, ng/mL		14.12 (3.01, 46.54)	13.45 (2.87, 46.50)	20.27 (7.86, 49.95)	0.055
NT-proBNP, pg/mL		2011.1 (1020.0, 4075.5)	1913.2 (984.9, 3835.0)	3779.6 (2280.5, 6881.7)	<0.001
hs-CRP, mg/L		26.40 (9.10, 71.00)	25.60 (8.70, 67.15)	33.60 (11.43, 113.50)	0.046
Na^+^, mmol/L		139.77 ± 3.30	139.82 ± 3.18	139.31 ± 4.23	0.220
K^+^, mmol/L		3.92 ± 0.48	3.91 ± 0.49	3.99 ± 0.47	0.160
Ca^+^, mmol/L		2.24 ± 0.14	2.24 ± 0.14	2.25 ± 0.15	0.403
Aspirin, n (%)		684 (94.34)	619 (94.50)	65 (92.86)	0.768
P_2_Y_12_ inhibitors, n (%)		712 (98.21)	642 (98.02)	70 (100.00)	0.474
β-blockers, n (%)		603 (83.17)	542 (82.75)	61 (87.14)	0.350
Statins, n (%)		697 (96.14)	629 (96.03)	68 (97.14)	0.894
ACEI/ARB, n (%)		396 (54.62)	356 (54.35)	40 (57.14)	0.656
Left atrial diameter, mm		39.30 ± 6.63	39.20 ± 6.64	40.31 ± 6.58	0.181
LVEF, %		51.44 ± 6.99	51.74 ± 6.69	48.70 ± 8.96	0.007
Killip, n (%)					0.004
	I	615 (84.83)	565 (86.26)	50 (71.43)	
	II	48 (6.62)	40 (6.11)	8 (11.43)	
	III	21 (2.90)	15 (2.29)	6 (8.57)	
	IV	41 (5.66)	35 (5.34)	6 (8.57)	
Length of stay, days		5.56 ± 2.48	5.51 ± 2.38	6.03 ± 3.26	0.203
LAD, n (%)		364 (50.21)	336 (51.30)	28 (40.00)	0.072
LCX, n (%)		92 (12.69)	81 (12.37)	11 (15.71)	0.424
RCA, n (%)		249 (34.34)	221 (33.74)	28 (40.00)	0.295
LM, n (%)		20 (2.76)	17 (2.60)	3 (4.29)	0.662

BMI, body mass index; PVD, peripheral vascular disease; TC, total cholesterol; LVEF, left ventricular ejection fraction; SBP, systolic blood pressure; DBP, diastolic blood pressure; LAD, left anterior descending; LCX, left circumflex artery; RCA, right coronary artery; LM, left main; ACEI, angiotensin-converting-enzyme inhibitor; ARB, angiotensin II receptor blocker; HDL-C, high-density lipoprotein cholesterol; LDL-C, low-density lipoprotein cholesterol; hs-CRP, high sensitivity C-reactive protein; TNI, troponin I; NT-proBNP, N-terminal pro-B-type natriuretic peptide; HF, heart failure.

### 3.2 Association Between HAVOC Score and NOAF in Patients With STEMI

**Fig. 2.**
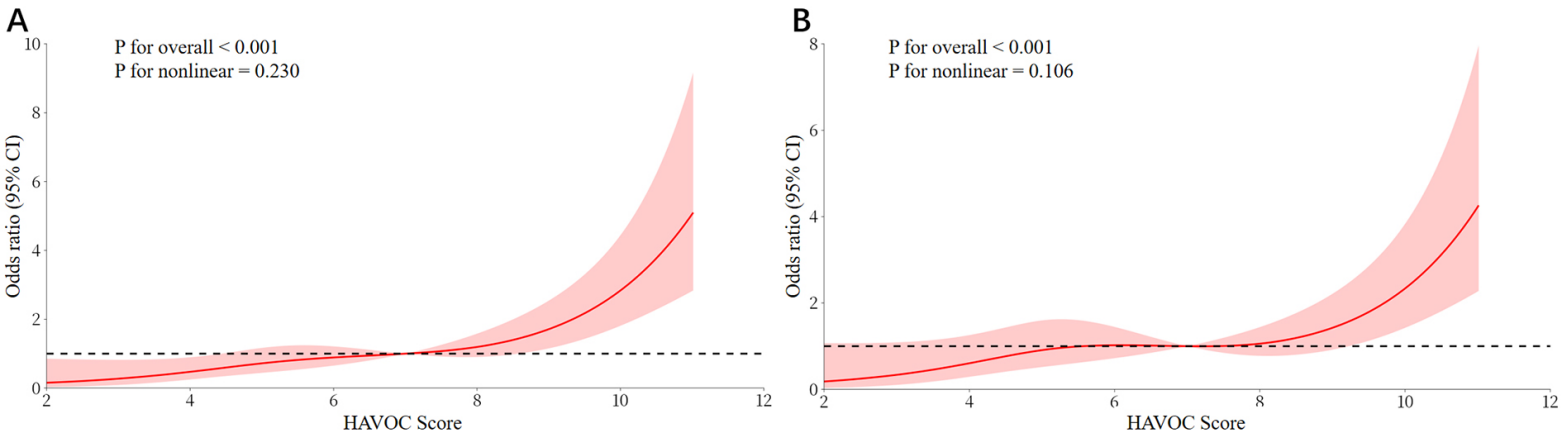
**The RCS models for HAVOC score and NOAF**. (A) Unadjusted dose-response curve showing the association between HAVOC score and risk of NOAF. (B) Adjusted dose-response curve after controlling for the risk factors.

Univariate logistic regression analysis indicated that the HAVOC Score, HF, age, LVEF, Killip class >1, hs-CRP, and NT-proBNP were associated with NOAF (**Supplementary Table 1**). These variables (excluding the component variables of the HAVOC Score), along with the left atrial diameter and left anterior descending artery (LAD) were incorporated into the multivariate analysis. The analysis indicated that the HAVOC score (odds ratio (OR) = 1.42; 95% confidence interval (CI): 1.28–1.59), LVEF, and LAD were independently related to the occurrence of NOAF (Table 2). The RCS analysis revealed a linear dose-response relationship between the HAVOC Score and NOAF (*p* for nonlinear >0.05, *p* for overall <0.001) (Fig. 2).

**Table 2.** **Multivariate regression analysis for NOAF**.

Variables	β (SE)	OR (95% CI)	*p*
HAVOC Score	0.35 (0.06)	1.42 (1.28–1.59)	<0.001
LVEF, %	–0.04 (0.02)	0.96 (0.93–1.00)	0.031
LAD, n (%)	–0.73 (0.27)	0.48 (0.28–0.83)	0.008

OR, odds ratio; CI, confidence interval; SE, standard error.

### 3.3 The Predictive Efficiency of HAVOC Score for NOAF in STEMI

**Fig. 3.**
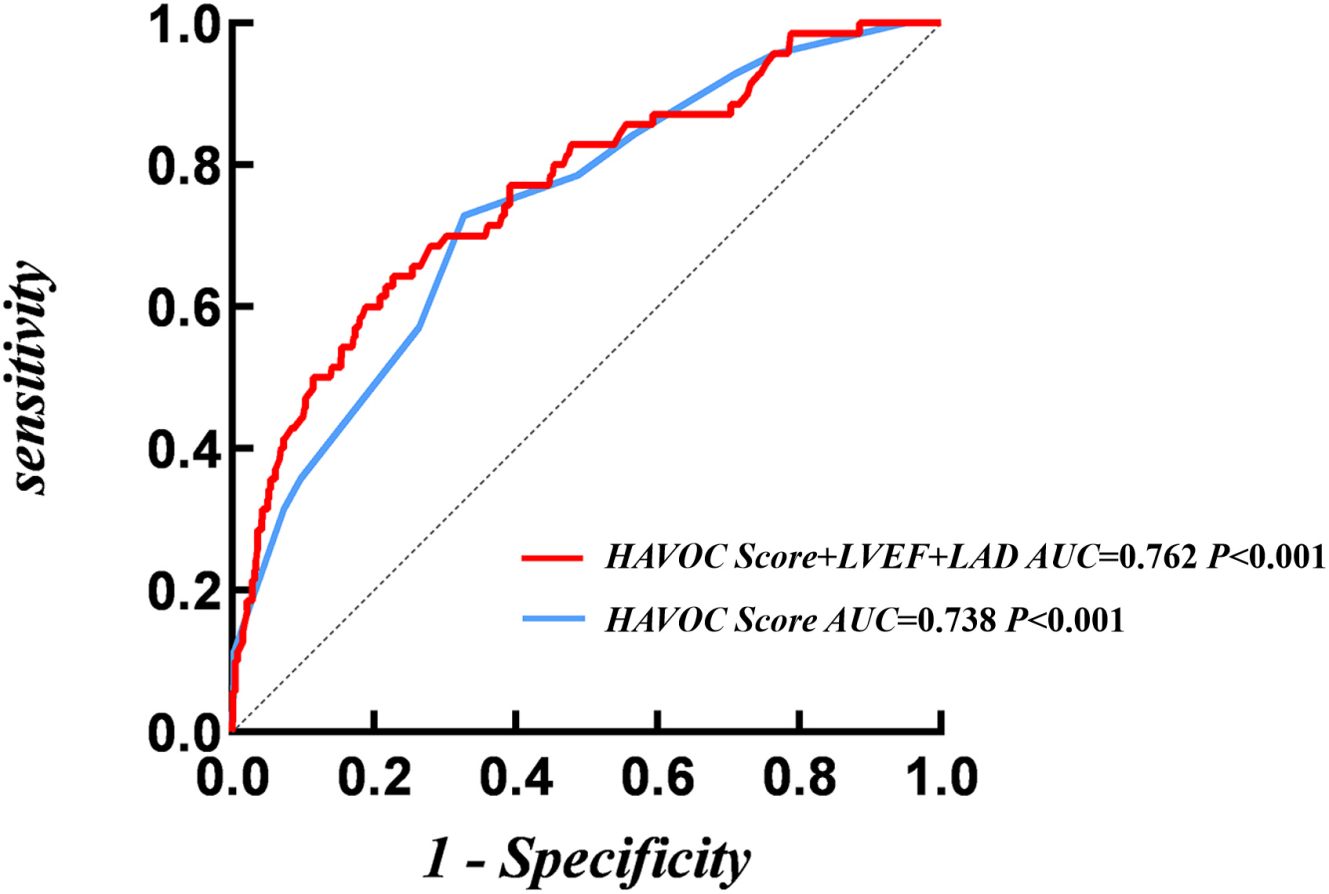
**ROC analysis of HAVOC score for NOAF**.

The ROC curve showed that the AUC of the HAVOC Score was 0.738 (95% CI: 0.677–0.798, *p* < 0.001), with a cut-off value of 5.50, the corresponding specificity was 0.673, and the sensitivity was 0.729. Variables with statistical significance in the multivariate logistic regression were incorporated to construct a new risk prediction model, which included the HAVOC Score, LVEF, and LAD. The ROC analysis of this new model showed an AUC of 0.762 (95% CI: 0.700–0.824, *p* < 0.001) (Fig. 3, Table 3).

**Table 3.** **ROC curve analysis for NOAF**.

	AUC	95% CI	*p*	Cut-off	Sensitivity	Specificity
HAVOC Score	0.738	0.677–0.798	<0.001	5.50	0.729	0.673
HAVOC Score + LVEF + LAD	0.762	0.700–0.824	<0.001	-	0.643	0.773

AUC, area under the curve; ROC, receiver operating characteristic.

The results of IDI and NRI revealed that the NRI was 0.353 (95% CI: 0.114–0.592, *p* = 0.004), and the IDI was 0.024 (95% CI: 0.006–0.041, *p* = 0.008). These findings suggested that the new model could significantly enhance the discriminatory ability of the HAVOC Score for identifying NOAF (Table 4).

**Table 4.** **Discrimination accuracy and reclassification of HAVOC for NOAF**.

	NRI		IDI	
	Estimate (95% CI)	*p*	Estimate (95% CI)	*p*
LVEF + LAD	Reference	-	Reference	-
HAVOC Score + LVEF + LAD	0.353 (0.114–0.592)	0.004	0.024 (0.006–0.041)	0.008

IDI, integrated discrimination improvement.

### 3.4 Sex Subgroup Analysis

Given the relatively small number of female participants, the subgroup analysis was restricted to male patients. In the univariate regression analysis, Na^+^, Ca^+^, HF, Killip class >1, hs-CRP, NT-proBNP, age, LVEF, HAVOC Score, heart rate, diastolic blood pressure, and low-density lipoprotein cholesterol were significantly associated with NOAF (**Supplementary Table 2**). After adjustment for these variables (excluding the components of the HAVOC Score) and for LAD and left atrial diameter, multivariate regression analysis confirmed that the HAVOC Score (OR = 1.31, 95% CI: 1.12–1.53) was independently associated with NOAF (Table 5).

**Table 5.** **Multivariate regression analysis for NOAF in sex subgroup analysis**.

Variables	β (SE)	OR (95% CI)	*p*
HAVOC Score	0.27 (0.08)	1.31 (1.12–1.53)	<0.001
LVEF, %	–0.06 (0.02)	0.95 (0.90–0.99)	0.016
NT-proBNP, pg/mL	0.92 (0.44)	2.51 (1.06–5.99)	0.038

LVEF, left ventricular ejection fraction; NT-proBNP, N-terminal pro-brain natriuretic peptide.

## 4. Discussion

This study is the first to evaluate the association between the HAVOC Score and in-hospital NOAF in STEMI patients undergoing PCI. We found that higher HAVOC values independently predicted NOAF, the risk increased in an approximately linear dose–response manner with increasing scores.

A recent report found that almost 70% of all AF patients have underlying CAD and that management of underlying CAD often results in a reduced burden of AF [17]. The impact of CAD on endothelial dysfunction can be another factor responsible for this effect as it results in inflammation which directly promotes AF [18, 19, 20]. NOAF following PCI in patients with STEMI represents a crucial clinical issue that significantly impacts patient outcomes [3, 4, 5, 6]. Although anticoagulant therapy has the potential to reduce the risk of death, it necessitates a careful balance against the risk of bleeding [7]. Despite existing studies having clearly defined the clinical characteristics and prognostic implications of NOAF, there is still a need for further exploration regarding the generalizability of risk models across different populations and the clinical translation of novel biomarkers.

The HAVOC Score, a novel assessment tool, includes multiple risk factors associated with AF. In patients with embolic stroke of undetermined source, a low HAVOC Score has been demonstrated to be associated with a low incidence of AF [8]. Among patients who have experienced cryptogenic stroke or transient ischemic attack, different HAVOC Scores can be utilized to determine varying levels of the risk of AF [9]. In another study focusing on patients with cryptogenic stroke, the HAVOC Score has proven capable of accurately identifying individuals at risk of AF during long-term monitoring [10]. Research on the HAVOC Score in patients with STEMI is still in its initial stage. Our study reveals that the HAVOC Score was independently associated with NOAF. In addition, we found a linear dose-response relationship between the HAVOC Score and NOAF. Hypertension can lead to structural and electrophysiological remodeling of the atrium through pressure overload [21]. After STEMI, myocardial ischemia further exacerbates the atrial load. In patients with hypertension, the process of atrial remodeling is accelerated, significantly increasing the risk of NOAF [22, 23]. It is well-known that myocardial fibrosis and autonomic nervous system dysfunction are the core mechanisms underlying the development of cardiovascular diseases in elderly patients [24]. In elderly patients, there is an increased apoptosis of atrial myocytes, and fibrous connective tissue replaces normal myocardium, resulting in a decrease of atrial conduction velocity and an increase in the dispersion of the refractory period [25, 26]. Valvular heart disease can cause diseases through hemodynamic disorders and abnormal atrial mechanical stress [27, 28]. Peripheral vascular disease is associated with systemic atherosclerosis and endothelial dysfunction, both of which are important risk factors promoting AF [29, 30, 31]. In obese patients, excessive accumulation of adipose tissue leads to insulin resistance and increased leptin levels, activating the RAS and the sympathetic nervous system, and inducing apoptosis and fibrosis of atrial myocytes. Inflammatory factors secreted by visceral fat directly participate in myocardial remodeling, leading to conduction heterogeneity [32, 33]. After STEMI, disorders of myocardial energy metabolism in obese patients may delay the repair process, reducing the tolerance of atrial myocytes to ischemic injury and significantly increasing the risk of NOAF [13]. HF has been proven in various diseases to be an important inducer of AF through atrial dilation and neurohumoral activation [34, 35]. In contrast to other diseases, STEMI patients often suffer from extremely severe myocardial ischemia, which can directly affect atrial function. During acute STEMI, ischemic injury can directly activate oxidative stress and the inflammatory response in atrial myocytes, inducing atrial arrhythmias [36]. The blood supply to the atrium is mainly provided by the right coronary artery (RCA) and the left circumflex artery (LCX) [37]. Therefore, when the culprit vessel is the RCA or LCX, it is often accompanied by atrial ischemia, increasing the risk of AF. In contrast, when the culprit lesion involves the LAD, the risk or extent of atrial ischemia may be lower, which may explain the lower incidence of NOAF observed in patients with LAD-related myocardial infarction. In our study, the LAD was demonstrated to be an independent protective factor for AF. The previous study has demonstrated a close association between C-reactive protein (CRP) levels and adverse outcomes in AF [38]. In a previous study, CRP was also identified as an independent risk factor for NOAF in patients with STEMI [39]. In contrast, although our study demonstrated that CRP levels were significantly elevated in the NOAF group, CRP was not an independent factor for NOAF after adjustment for confounding factors. This discrepancy may be partly explained by the inclusion of the HAVOC Score in our multivariable model, as several of its components are closely related to inflammation. The association between CRP and NOAF in STEMI patients remains controversial [40]. Therefore, further studies with more targeted and mechanism-based designs are warranted to clarify this relationship.

In our study, when the HAVOC Score is combined with LVEF and LAD, the results of IDI and NRI, suggest a significant enhancement in the ability to identify NOAF in STEMI patients after PCI. Impaired cardiac function and myocardial ischemia, which distinguish STEMI from other diseases, are key contributors to NOAF. Therefore, our study may provide more information beyond the HAVOC Score for the risk stratification of NOAF in STEMI patients. In future research, it may be advisable to consider incorporating LVEF and LAD into the HAVOC Score to develop and validate a scoring model for NOAF in STEMI patients, enabling more accurate identification of high-risk patients and achieving individualized risk stratification. This integrative approach may help clinicians to refine post-PCI management strategies, such as closer rhythm monitoring and optimization of cardioprotective therapy. In addition, to enhance clinical applicability, future studies could perform risk stratification based on the HAVOC Score, dividing patients into low-, moderate-, and high-risk groups. However, the larger, prospective cohort studies, are still needed to validate the corresponding cut-off values and the predictive performance of this stratification approach.

### Limitations

First, this is a single-center retrospective study, and there may be some unavoidable biases. Some potential confounders that may influence the risk of AF, such as infarct size and delay in reperfusion, were not controlled for; therefore, our findings should be interpreted in this context. Second, our study focused on the events of NOAF during the hospitalization period. Whether there are differences in the long-term prognosis of these patients and whether the HAVOC Score can predict NOAF in the long term requires further clarification. Third, this study was conducted in a single-center Chinese population, which may limit the generalizability of the findings to other ethnic or demographic groups. Fourth, our ascertainment of AF was based on the standard ECG and at least 72 hours of ECG monitoring, rather than continuous rhythm surveillance covering the entire hospital stay. This approach may have led to a non-differential misclassification of the outcome by potentially under-detecting cases of paroxysmal or subclinical AF.

## 5. Conclusions

Higher HAVOC Scores were independently associated with a greater risk of in-hospital NOAF among STEMI patients after PCI, showing an approximately linear dose–response relationship. These findings highlight the HAVOC Score as a practical tool for early identification of patients at increased risk for NOAF and may result in improved monitoring and preventive strategies in clinical practice.

## Availability of Data and Materials

The datasets used and analyzed during the present study are available from the corresponding author on reasonable request.

## Supplementary Material

Supplementary material associated with this article can be found, in the online version, at https://doi.org/10.31083/RCM47061.
