# Supplementary material for: Association Between HAVOC Score and New-Onset Atrial Fibrillation in Patients With ST-Segment Elevation Myocardial Infarction
Source: Rev Cardiovasc Med. 2026 Feb 25;27(2):47061. doi: 10.31083/RCM47061 (PMC12960014; doi:10.31083/RCM47061)
Supplement: Supplementary file 1 [file 2153-8174-27-2-47061-s1.zip › Supplementary Material.docx]

### Supplementary Table 1**. Univariate Regression Analysis for NOAF**

| Variables | OR (95%CI) | *P* |
| --- | --- | --- |
| Age, years | 1.11 (1.08 ~ 1.14) | <.001 |
| Male, n (%) | 0.64 (0.38 ~ 1.07) | 0.087 |
| BMI, kg/m^2^ | 1.03 (0.96 ~ 1.10) | 0.401 |
| Heart rate, bpm | 1.01 (0.99 ~ 1.03) | 0.185 |
| SBP, mmHg | 1.00 (0.99 ~ 1.01) | 0.697 |
| DBP, mmHg | 0.99 (0.97 ~ 1.01) | 0.186 |
| Smoking, n (%) | 0.85 (0.52 ~ 1.41) | 0.532 |
| HF, n (%) | 3.84 (2.13 ~ 6.93) | <.001 |
| Valvular disease, n (%) | 1.41 (0.77 ~ 2.59) | 0.265 |
| PVD, n (%) | 1.13 (0.62 ~ 2.05) | 0.695 |
| Hypertension, n (%) | 1.57 (0.96 ~ 2.57) | 0.074 |
| Diabetes, n (%) | 1.37 (0.79 ~ 2.37) | 0.262 |
| Stroke, n (%) | 1.14 (0.54 ~ 2.39) | 0.727 |
| HAVOC Score | 1.43 (1.29 ~ 1.59) | <.001 |
| TNI, ng/mL | 1.00 (0.99 ~ 1.01) | 0.982 |
| NT-proBNP, pg/mL | 3.75 (2.20 ~ 6.42) | <.001 |
| hs-CRP, mg/L | 1.01 (1.01 ~ 1.01) | 0.006 |
| TC, mmol/L | 0.85 (0.66 ~ 1.09) | 0.190 |
| Triglycerides, mmol/L | 1.17 (0.94 ~ 1.46) | 0.159 |
| LDL-C, mmol/L | 0.75 (0.56 ~ 1.01) | 0.059 |
| HDL-C, mmol/L | 0.35 (0.11 ~ 1.10) | 0.073 |
| Na^+^, mmol/L | 0.96 (0.89 ~ 1.03) | 0.220 |
| K^+^, mmol/L | 1.38 (0.88 ~ 2.17) | 0.162 |
| Ca^+^, mmol/L | 2.14 (0.36 ~ 12.63) | 0.403 |
| Aspirin, n (%) | 0.76 (0.29 ~ 1.99) | 0.572 |
| β-blockers, n (%) | 1.41 (0.68 ~ 2.93) | 0.352 |
| Statins, n (%) | 1.41 (0.33 ~ 6.05) | 0.648 |
| ACEI/ARB, n (%) | 1.12 (0.68 ~ 1.84) | 0.656 |
| Left atrial diameter, mm | 1.02 (0.99 ~ 1.06) | 0.181 |
| LVEF, % | 0.94 (0.91 ~ 0.98) | <.001 |
| KILLIP >1, n (%) | 2.51 (1.43 ~ 4.41) | 0.001 |
| LM, n (%) | 1.68 (0.48 ~ 5.88) | 0.417 |
| LAD, n (%) | 0.63 (0.38 ~ 1.05) | 0.074 |
| LCX, n (%) | 1.32 (0.67 ~ 2.62) | 0.425 |
| RCA, n (%) | 1.31 (0.79 ~ 2.17) | 0.296 |

BMI, body Mass Index; PVD, peripheral vascular disease; TC, total cholesterol; LVEF, left ventricular ejection fraction; SBP, systolic blood pressure; DBP, diastolic blood pressure; LAD, left anterior descending; LCX, left circumflex artery; RCA, right coronary artery; LM, left main; ACEI, angiotensin-converting-enzyme inhibitor; ARB, angiotensin II receptor blocker; HDL-C, high-density leptin cholesterol; LDL-C, low-density leptin cholesterol; hs-CRP, high sensitivity C-reactive protein; TNI, troponin I; NT-proBNP, N-terminal pro-B-type natriuretic peptide; HF, heart failure.

### Supplementary Table 2**. Univariate Regression Analysis for NOAF** i**n Sex Subgroup Analysis**

| Variables | OR (95%CI) | *P* |
| --- | --- | --- |
| Age, years | 1.11 (1.07 ~ 1.15) | <.001 |
| BMI, kg/m^2^ | 0.99 (0.91 ~ 1.09) | 0.906 |
| Heart rate, bpm | 1.02 (1.01 ~ 1.04) | 0.048 |
| SBP, mmHg | 0.99 (0.97 ~ 1.00) | 0.091 |
| DBP, mmHg | 0.97 (0.95 ~ 0.99) | 0.032 |
| Smoking, n (%) | 0.88 (0.48 ~ 1.62) | 0.683 |
| HF, n (%) | 5.63 (2.65 ~ 11.95) | <.001 |
| Valvular disease, n (%) | 1.23 (0.57 ~ 2.64) | 0.604 |
| PVD, n (%) | 1.09 (0.52 ~ 2.27) | 0.816 |
| Hypertension, n (%) | 0.88 (0.47 ~ 1.67) | 0.704 |
| Diabetes, n (%) | 0.75 (0.34 ~ 1.66) | 0.483 |
| Stroke, n (%) | 1.80 (0.76 ~ 4.26) | 0.183 |
| HAVOC Score | 1.44 (1.27 ~ 1.64) | <.001 |
| TNI, ng/mL | 1.00 (1.00 ~ 1.01) | 0.343 |
| NT-proBNP, pg/mL | 7.15 (3.51 ~ 14.59) | <.001 |
| hs-CRP, mg/L | 1.01 (1.01 ~ 1.01) | 0.007 |
| TC, mmol/L | 0.79 (0.57 ~ 1.10) | 0.167 |
| Triglycerides, mmol/L | 1.20 (0.94 ~ 1.54) | 0.151 |
| LDL-C, mmol/L | 0.64 (0.43 ~ 0.95) | 0.026 |
| HDL-C, mmol/L | 0.32 (0.07 ~ 1.49) | 0.147 |
| Na^+^, mmol/L | 0.91 (0.84 ~ 0.99) | 0.034 |
| K^+^, mmol/L | 1.56 (0.88 ~ 2.74) | 0.127 |
| Ca^+^, mmol/L | 16.58 (1.52 ~ 180.95) | 0.021 |
| Aspirin, n (%) | 0.83 (0.19 ~ 3.70) | 0.807 |
| β-blockers, n (%) | 1.06 (0.46 ~ 2.46) | 0.894 |
| Statins, n (%) | 1.21 (0.16 ~ 9.50) | 0.853 |
| ACEI/ARB, n (%) | 1.12 (0.60 ~ 2.09) | 0.719 |
| Left atrial diameter, mm | 1.02 (0.97 ~ 1.06) | 0.469 |
| LVEF, % | 0.92 (0.88 ~ 0.95) | <.001 |
| KILLIP >1, n (%) | 2.87 (1.43 ~ 5.78) | 0.003 |
| LM, n (%) | 1.36 (0.30 ~ 6.11) | 0.688 |
| LAD, n (%) | 0.79 (0.43 ~ 1.45) | 0.445 |
| LCX, n (%) | 0.93 (0.38 ~ 2.27) | 0.865 |
| RCA, n (%) | 1.29 (0.68 ~ 2.42) | 0.432 |

BMI, body Mass Index; PVD, peripheral vascular disease; TC, total cholesterol; LVEF, left ventricular ejection fraction; SBP, systolic blood pressure; DBP, diastolic blood pressure; LAD, left anterior descending; LCX, left circumflex artery; RCA, right coronary artery; LM, left main; ACEI, angiotensin-converting-enzyme inhibitor; ARB, angiotensin II receptor blocker; HDL-C, high-density leptin cholesterol; LDL-C, low-density leptin cholesterol; hs-CRP, high sensitivity C-reactive protein; TNI, troponin I; NT-proBNP, N-terminal pro-B-type natriuretic peptide; HF, heart failure.
